# Morphofunctional analysis of the retina in patients with type 1 diabetes without complications after 30 years of disease

**DOI:** 10.1038/s41598-019-57034-1

**Published:** 2020-01-14

**Authors:** Riccardo Sacconi, Francesca Lamanna, Enrico Borrelli, Giacomo Mulinacci, Marco Casaluci, Francesco Gelormini, Adriano Carnevali, Lea Querques, Gianpaolo Zerbini, Francesco Bandello, Giuseppe Querques

**Affiliations:** 1 Department of Ophthalmology, University Vita-Salute, IRCCS Ospedale San Raffaele, Milan, Italy; 2 Complications of Diabetes Unit, Division of Metabolic and Cardiovascular Sciences, San Raffaele Scientific Institute, Milan, Italy

**Keywords:** Retinal diseases, Diabetes

## Abstract

There is a lack of studies evaluating the sub-clinical retinal changes in patients with long-term type 1 diabetes mellitus (T1DM) and without history of systemic/ocular complications. The aim of this cross-sectional study was to investigate sub-clinical structural and/or vascular retinal changes in patients with long-term (≥30 years) T1DM and without systemic/ocular complications (“happy few” patients) using structural optical coherence tomography (OCT), OCT-angiography and microperimetry. Twelve eyes of 12 consecutive T1DM patients (mean age 52 ± 12 years, mean duration of disease 35 ± 3 years, mean HbA1c level 7.3 ± 2.8%), without micro/macrovascular complications associated with long-standing T1DM, and twelve healthy subjects were consecutively included. No statistically significant differences were disclosed comparing patients and controls for age, sex, best-corrected visual acuity, central macular thickness, and choroidal thickness. Using OCT-angiography, we did not find any significant difference in foveal avascular zone area, perfusion density, vessel length density, and tortuosity. Moreover, no significant differences were disclosed in retinal nerve fiber layer and ganglion cell complex thickness using structural OCT. No differences were disclosed in retinal sensitivity by microperimetry. New diagnostic tools are able to confirm the presence of a particular population of patients with type 1 diabetes who have been completely spared from diabetic retinal complications. The finding of these “happy few” patients could help us to better understand and target future treatments for diabetes.

## Introduction

Diabetic retinopathy (DR) is the most common microvascular complication of diabetes and it develops in the majority of patients with long-standing type 1 diabetes mellitus (T1DM)^1^. For this reason, the American Diabetes Association (ADA) recommended a comprehensive ocular examination within 5 years after T1DM diagnosis in order to detect early signs of DR and to manage it promptly^2^. Retinal microaneurysms may represent the first visible clinical sign of DR and are secondary to small outpouchings of the retinal capillaries.

Recently, new imaging technologies have enabled early identification of retinal structural and functional changes, even before DR is clinically evident. Optical coherence tomography angiography (OCT-A), a non-invasive dye-free imaging modality, provides a highly detailed view of the retinal and choroidal vasculature. Nowadays, optimized OCT-A algorithms may be used to detect early retinal and choriocapillaris (CC) microvascular flow alterations, which may be evident even before the DR presentation and may potentially result in irreversible retinal damage. As an example, De Carlo *et al*.^3^ revealed OCT-A ability to detect foveal avascular zone (FAZ) remodeling and retinal capillary nonperfusion in T1DM patients without DR. Furthermore, both our group^4^ and Simonett *et al*.^5^ reported a significantly decreased perfusion density (PD) in the deep retinal vascular complex (DVC) in T1DM patients without evidence of DR signs at fundus examination.

Developments of high resolution structural optical coherence tomography (OCT) technology have allowed us to identify thinning of the inner retinal layers in T1DM patients without DR compared to healthy controls, suggesting that a gradual loss of neurons may occur, even in the absence of visible signs of vascular retinopathy^6,7^. Notably, Vujosevic *et al*.^8^ demonstrated a significant inverse correlation between perifoveal capillary loss in superficial capillary plexus (SCP) and inner retinal layer thickness in patients with DM and no clinical signs of DR. Since retinal thickness is decreased in pubescent T1DM children without DR and correlates with HbA1C level, some authors suggested that OCT can be considered a tool for early detection of retinal abnormalities in patients with diabetes^9^.

Previously, retinal functional abnormalities using microperimetry were reported in patients without clinically visible funduscopic retinopathy^10^. Nittala and coworkers^11^ displayed a reduced macular sensitivity in patients with diabetes and no DR compared to healthy controls, thus suggesting that diabetes might affect visual function before clinically signs of DR^12,13^.

Recent evidence suggests that microvascular complications related to diabetes may spare a group of patients affected by diabetes. In particular, the Medalist study^14^ demonstrated that a small subgroup of patients (“happy few” patients with type 1 diabetes without complications) with more than 50 years of type 1 diabetes do not display systemic microvascular complications. This evidence may suggest that some mechanisms in patients with diabetes may drive a “resistance” to develop microvascular complications^14^.

To our knowledge, there is a lack of studies evaluating sub-clinical retinal changes in patients with long-term T1DM and without evidence or history of systemic and ocular complications. Therefore, the aim of this study was to analyze morphofunctional parameters of the retina in these patients, in order to assess the presence of sub-clinical retinal alterations in the “happy few” patients.

## Methods

### Study participants

Patients presenting with diagnosis of type 1 diabetes for more than 30 years were consequently enrolled between January 2018 and June 2018 at the Medical Retina and Imaging Unit of the Department of Ophthalmology, University Vita-Salute, San Raffaele Hospital in Milan from a pool of patients followed at Endocrinology Department of the same hospital. The study adhered to the 1964 Helsinki declaration and its later amendments. Informed consent was obtained from all individual participants included and it was approved by the Local Ethics Committee of San Raffaele Hospital, Milan, Italy.

Inclusion criteria for the study group were: (1) history of T1DM for at least 30 years before enrollment, (2) absence of any signs of DR at fundus examination, and (3) absence of evidence or history of systemic diabetes-associated complications, including nephropathy, end-stage renal disease, peripheral neuropathy, peripheral vascular disease, gangrene, amputation, angina, myocardial infarction, chronic heart failure, stroke, and transient ischemic attack. The diagnosis of T1DM was based on the National Institute for Health and Care Excellence (NICE) guidelines.

[available on https://www.nice.org.uk/guidance/ng18/chapter/1-Recommendations#diagnosis].

Exclusion criteria included: (1) presence or history of any other retinal disease (e.g. retinal vascular diseases, vitreoretinal diseases, history of central serous retinopathy or macular dystrophies), (2) optic nerve disease, (3) any previous eye surgical intervention or laser photocoagulation, and (4) absence of clear ocular media, adequate pupillary dilation and fixation to permit high-quality imaging, (5) refractive error greater than +5 diopters (D) or −6D of sphere or ±3D of cylinder.

If both eyes of a patient were eligible, only one eye was randomly chosen flipping a coin and included.

During the period of patient recruitment, an additional cohort of healthy control subjects matched for age and sex were also recruited. All healthy subjects had no ocular disorders and were visited by a senior author (GQ) in the Department of Ophthalmology, University Vita-Salute, San Raffaele Hospital in Milan.

Patients with type 1 diabetes mellitus and control subjects underwent a complete ophthalmologic evaluation, including: assessment of distance best-corrected visual acuity (BCVA) using Snellen charts, dilated slit-lamp anterior segment and fundus biomicroscopy, structural spectral domain-OCT (SD-OCT, Spectralis, Heidelberg Engineering, Heidelberg, Germany), Swept-Source OCT-A (PLEX Elite 9000, Carl Zeiss Meditec, Inc., Dublin, USA), assessment of retinal sensitivity by microperimetry (MP-1, Nidek Technologies, Padova, Italy).

### OCT-A image acquisition and analysis

In all patients, a scanning area of 3 × 3 mm was adopted (300 A-scans per B-scan repeated four times at each of the 300 B-scan positions), centered on the foveal area. FastTrac motion correction software was used while the images were acquired to reduce motion artifacts. To be included in the analysis, we considered only OCT-A volume scan sets with a signal strength index (SSI) >7.

**Figure 1:**
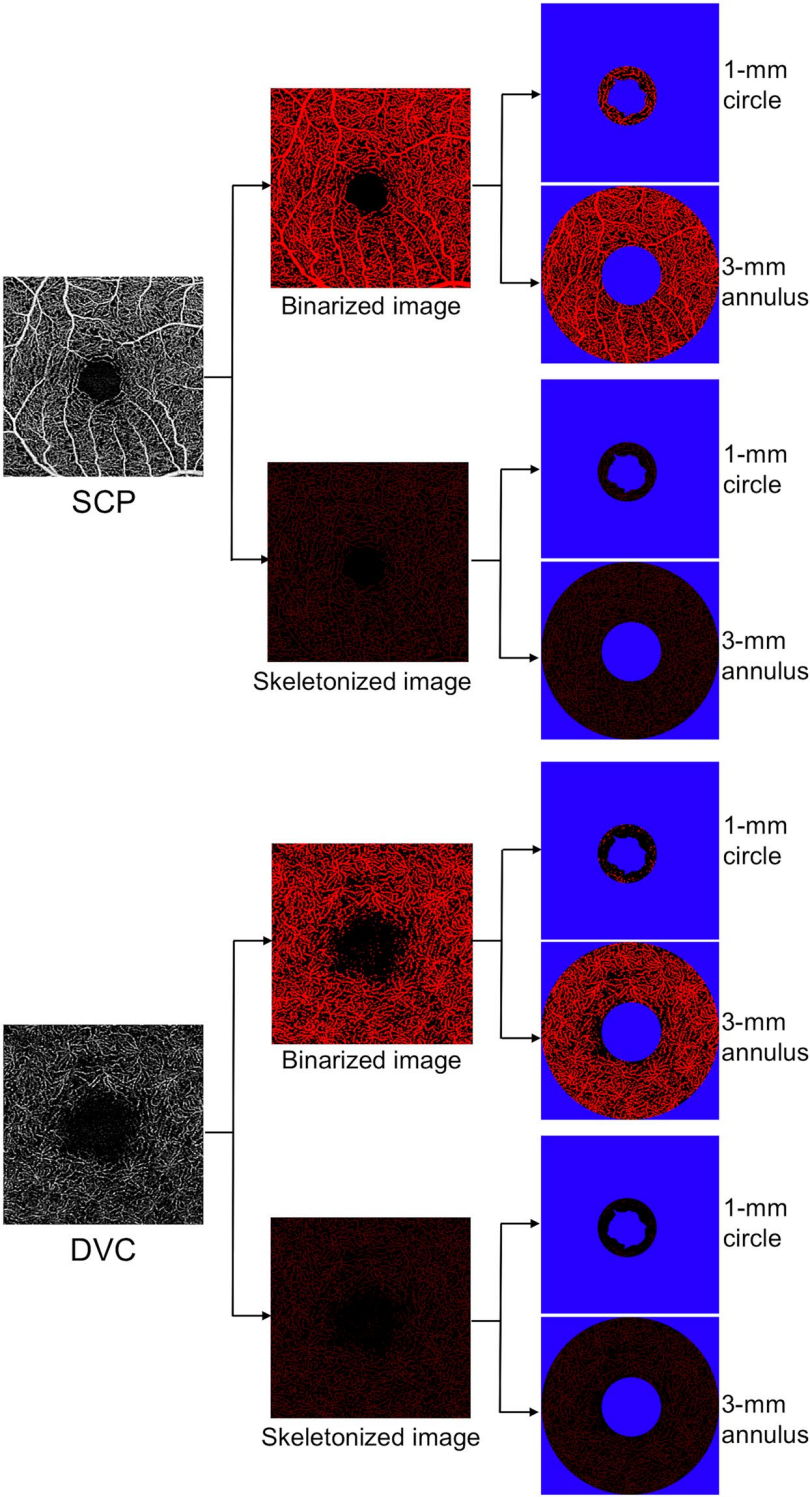
Representation of the algorithm used to process superficial capillary plexus and deep vascular complex images. The images of the superficial capillary plexus (SCP) and deep vascular complex (DVC) were first imported in ImageJ software. These images were binarized and the obtained images were used to test the perfusion density, which was calculated as a unitless proportion of the number of pixels over the threshold (red pixels in the binarized images) divided by the total number of pixels in the analyzed region of interest (ROI) after blue pixels exclusion. The binarized SCP and DVC images were also skeletonized, in order to obtain a slab in which vessels are visualized as trace of 1 pixel in width. These images were thus used to investigate the vessel length density, which was defined as the total length of perfused vasculature divided by the total number of pixels in the analyzed ROI after blue pixels exclusion, and vessel tortuosity. All images were investigated in two different ROI after foveal avascular zone exclusion (colored in pure blue): the foveal region (1-mm circle centered on the fovea) and parafoveal area (inside a circular annulus between 1 mm and 3 mm centered on the fovea). In detail, images included in Fig. 1 are of a healthy patient, but the same algorithm was used for patients with type 1 diabetes.

**Figure 2:**
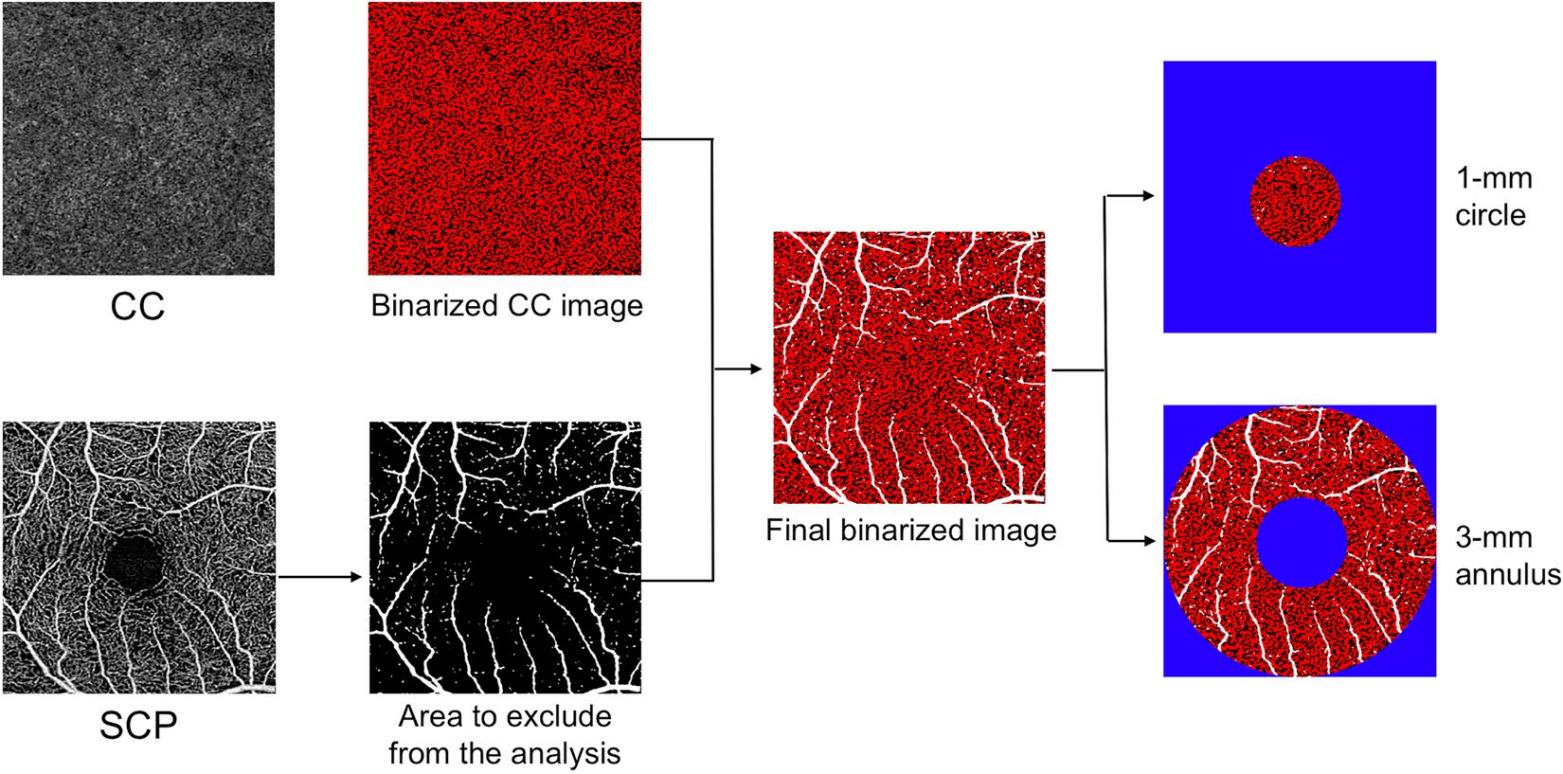
Representation of the algorithm used to process choriocapillaris images. The images of choriocapillaris (CC) were first imported in ImageJ software and a “Phansalkar” threshold was applied to binarize the CC images. Pixels with an intensity over the applied threshold are marked in “red” in the final image and those pixels falling below the threshold are marked in “black”. In order to precisely identify and mask the superficial vessels (white in the final image), we used the superficial capillary plexus en face optical coherence tomography angiography image, which was thresholded and “binarized”. Perfusion density was calculated as a unitless proportion of the red pixels divided by the total number of pixels in the analyzed region of interest (ROI) after blue and white pixels exclusion. All images were investigated in two different ROI: the foveal region (1-mm circle centered on the fovea) and parafoveal area (inside a circular annulus between 1 mm and 3 mm centered on the fovea). In detail, images included in Fig. 2 are of a healthy patient, but the same algorithm was used for patients with type 1 diabetes.

En face flow images were obtained using the inbuilt software of PLEX Elite (version 1.7.1.31492). Briefly, a fully-automated retinal layer segmentation algorithm was applied to the three-dimensional structural OCT data, in order to segment the SCP, DVC and CC slabs^15^. The inbuild software of PLEX Elite does not provide an optimized segmentation of the intermediate capillary plexus (ICP) and, for this reason, we analyzed the DVC, including both ICP and DCP as previously reported^16^. These segmentations were applied to OCT-A flow intensity data with maximum projection, to obtain vascular images. En face flow images were exported from the PLEX Elite using the “Export” function, resulting in images with 1024 × 1024 pixels for the 3 × 3 scan. All images were exported into ImageJ 1.50 (National Institutes of Health, Bethesda, Maryland, USA) software (Figs. 1 and 2). FAZ area was manually outlined by two trained graders (E.B. and A.C.) using the polygon selection tool in a full-retinal thickness, and its dimension was expressed as square millimeters (mm^2^). The mean measurement of the FAZ area between the two readers was considered for the statistical analysis. Perfusion density of SCP and DVC was calculated through Mean’s thresholding and binarization, according to previous studies (Fig. 1)^17,18^. Perfusion density of CC was calculated through Phansalkar’s thresholding (radius, 15 pixels) and binarization after removing from the analysis the CC directly beneath major superficial retinal vessels in order to eliminate potentially confounding shadow or projection artifacts (Fig. 2)^19^. CC slab was identified as a 20 μm thick starting 29 μm posterior to the RPE reference.

Each 3 × 3 image was investigated in two different areas: foveal region (inside a circle of 0.5 mm radius centered on the fovea) and parafoveal area (inside a circular annulus between 0.5 mm and 1.5 mm radius centered on the fovea). Perfusion density was calculated for SCP, DVC, and CC as the ratio between the red pixels and the total pixels after FAZ exclusion (Figs. 1 and 2).

Binarized images of SCP and DVC were then converted to skeletonized images, where vessels were reduced to a width of one pixel (Fig. 1). Skeletonized images were used to calculate vessel length density (VLD) as the ratio of the total skeleton area over the total scanned area^19^. Vessel tortuosity was calculated according to the method recently published by Lee Hyungwoo, *et al*.^20^.

### Structural SD-OCT measurements

For each patient, a detailed macular and peripapillary structural OCT was also acquired. Central macular thickness (CMT) was recorded with the Spectralis software (Heidelberg Eye Explorer, version 1.9.11.0 Heidelberg Engineering, Germany) in the central 1-mm-diameter circle (foveal area) and in a circular annulus between 0.5 mm and 1.5 mm radius centered on the fovea (parafoveal area) of the ETDRS thickness map. The Spectralis software was also used to obtain each retinal layer thickness, in particular macular and peripapillary retinal nerve fiber layer (RNFL) and macular ganglion cell complex (GCC).

In detail, as for macular RNFL and GCC, 1-mm-diameter central circle (C), 3-mm-diameter and 6-mm-diameter subfield of nasal (N), temporal (T), superior (S) and inferior (I) quadrants as defined by ETDRS were recorded and used for the analysis. With regard to 4.1-mm-diameter peripapillary RNFL, central (G) and subfield of nasal (N), nasal superior (NS), temporal superior (TS), temporal (T), temporal inferior (TI) and nasal inferior (NI) quadrants were recorded and used for the analysis.

To achieve a better visualization of the choroid, enhanced depth imaging (EDI) OCT was used in all macular acquisitions.

Choroidal thickness (ChT) was assessed by manually measuring the subfoveal distance between Bruch’s membrane interface and sclerochoroidal interface to identify the inner and outer boundaries of the choroid, respectively. A manual function was used because Spectralis OCT does not provide an automatic segmentation of the choroid. The subfoveal ChT was assessed by two trained graders (E.B. and A.C.) and mean measurement was considered for the statistical analysis.

### Microperimetry

Assessment of retinal sensitivity was performed using microperimetry following the same procedure used in a previously published paper^21^. In detail, before the examination, all patients underwent at least 5 mm pupil dilation with 1% tropicamide and 15-minute mesopic adaptation. For our clinical practice, during the first examination, all patients underwent a fast test only for learning. After that, the test was deleted and the patient started with the first recorded examination. In all patients, a customized grid of 33 Goldmann II stimuli, covering the central 10° (centered on the fovea), were presented in random order according to a 4-2-1 double-staircase strategy. The stimulus intensity ranged from 0 dB to 20 dB (0 dB corresponded to the strongest signal intensity of 127 cd/m^2^) in 1-dB steps, and the duration of each stimulus was 200 milliseconds.

The following parameters were recorded in each examination: mean retinal sensitivity (MS) of all analyzed central 10°, mean fixation percentage within the central 2° and 4° (centered on the fovea).

### Statistical analysis

Statistical analysis was performed using SPSS statistics software version 22.0 (SPSS Inc., IBM, Chicago, IL, USA). All quantitative data were expressed as mean ± standard deviation. Qualitative data were expressed as count and percentage. The intraclass correlation coefficient (ICC; 95% CI) was used to estimate the agreement between individual measurements from both readers. Categorical variables were analyzed using χ^2^ test. Continuous variables were tested for normal distributions, according to the Kolmogorov- Smirnov test. Comparisons of mean values between patients with type 1 diabetes without complications and control subjects were made using the Student’s t-test for independent samples. Also comparisons of mean values between patients under treatment with continuous subcutaneous insulin infusion (CSII) and with insulin injections, and between patients with and without systemic hypertension (HTN) were made using the Student’s t-test for independent samples. Correlations between MS with age and HbA1c level were assessed calculating Pearson’s correlation coefficients. A p-value ≤ 0.05 was considered to be statistically significant.

## Results

### Characteristics of subjects included in the analysis

Twelve eyes of 12 patients with long-term T1DM and without evidence or history of systemic and ocular complications and 12 healthy subjects with no prior ophthalmologic or medical history were included in this study. Six patients (50%) were under treatment using insulin pump therapy (CSII) whereas the other six patients were in treatment with mixed long-acting and short-acting insulin injections. Systemic HTN was recorded in 3 patients (25%) and was under control by medications.

The mean age of patients with type 1 diabetes without complications was 52 ± 12 years (median 49.5; range 40–76 years), and all patients were Caucasian (9 females and 3 males). The mean refractive error was −0.71 ± 1.30 D. Mean duration of the disease was 35 ± 3 years (range 30–40 years), and the mean HbA1c level was 7.3 ± 2.8% (range 6.2–8.3%).

Healthy subjects included were homogenous for age and sex: the mean age was 51 ± 9 years (median 55; range 37–60 years [p = 0.870]), with 9 females and 3 males (p = 1.00). Furthermore, healthy subjects did not show any significant difference in mean refractive error (mean refractive error was −0.08 ± 1.98 D, p = 0.380). Systemic HTN was recorded in 2 patients (17%) and was under control by medications.

All diabetic and control eyes were correctable to was 20/20 Snellen equivalent BCVA (Table 1).

**Table 1:** Demographic and clinical characteristics of eyes of patients with type 1 diabetes without complications and control eyes.

	Diabetic eyes (n = 12)	Control eyes (n = 12)	P value
Gender (male/female)	3/9	3/9	1.000*
DM duration (mean ± SD), years	35 ± 3	\	\
HbA1c, %	7.3 ± 2.8	\	\
Systemic HTN, n	3	2	0.615*
Eye (left/right)	6/6	7/5	0.682*
Age (mean ± SD), years	52 ± 12	51 ± 9	0.870^+^
CMT in foveal area (mean ± SD), µm	278 ± 20	269 ± 18	0.270^+^
CMT in parafoveal area (mean ± SD), µm	336 ± 17	339 ± 12	0.637^+^
Subfoveal ChT (mean ± SD), µm	292 ± 64	250 ± 42	0.068^+^

n, number; SD, standard deviation; DM, diabetes mellitus; HbA1C, haemoglobin A1c; HTN: hypertension, CMT: central macular thickness; ChT: choroidal Thickness.

*χ^2^ test; ^+^Student’s t test for independent samples.

### Structural OCT analysis

No significant difference between patients with type 1 diabetes without complications and controls was disclosed in CMT of foveal area (278 ± 20 µm [range 243–308 µm] and 269 ± 18 µm [range 234–294 µm] for diabetic and control eyes, respectively [p = 0.270]) and of the parafoveal area (336 ± 17 µm [range 303–365 µm] and 339 ± 12 µm [range 320–356 µm] for diabetic and control eyes, respectively [p = 0.637]). No significant difference was disclosed in the subfoveal ChT (292 ± 64 µm [range 187–395 µm] and 250 ± 42 µm [range 184–348 µm] for diabetic and control eyes, respectively [p = 0.068]) (Table 1). Interobserver variability between readers was excellent for subfoveal ChT measurements (ICC = 0.993 [0.985–0.997]).

Regarding the analysis of retinal layer thickness using structural SD-OCT, no significant differences were disclosed in the macular RFNL and GCC thickness comparing each 3-mm-diameter subfield and 6-mm-diameter subfield (C, S, I, N and T) of patients with type 1 diabetes without complications with the corresponding subfield of control subjects. In addition, no differences were found comparing each quadrant (G, N, NS, TS, T, TI and NI) of the peripapillary RNFL of the two groups (Table 2).

**Table 2:** Ganglion cell complex and retinal nerve fiber layer thickness analysis of eyes of patients with type 1 diabetes without complications compared with control group.

	Diabetic eyes (n = 12)	Control eyes (n = 12)	P value*
	*Mean* ± *SD*	*Mean* ± *SD*	
GCC thickness (µm)			
1-mm central circle	14.3 ± 3.9	13.4 ± 2.2	0.483
3-mm S subfield	51.0 ± 4.3	52.6 ± 3.4	0.330
6-mm S subfield	33.6 ± 3.3	35.8 ± 4.2	0.159
3-mm I subfield	49.6 ± 5.5	53.3 ± 4.3	0.083
6-mm I subfield	33.5 ± 2.5	34.1 ± 3.4	0.590
3-mm N subfield	50.0 ± 4.0	51.5 ± 4.3	0.389
6-mm N subfield	37.6 ± 3.3	38.0 ± 4.2	0.630
3-mm T subfield	44.3 ± 4.8	47.1 ± 4.8	0.148
6-mm T subfield	34.8 ± 3.8	35.3 ± 3.8	0.751
Macular RNFL thickness (µm)			
1-mm central circle	12.3 ± 1.9	12.0 ± 1.7	0.658
3-mm S subfield	23.9 ± 2.4	23.9 ± 2.0	1.000
6-mm S subfield	37.1 ± 5.2	35.7 ± 4.3	0.499
3-mm I subfield	23.7 ± 3.5	24.7 ± 2.1	0.407
6-mm I subfield	38.1 ± 5.1	39.0 ± 3.4	0.612
3-mm N subfield	20.3 ± 1.4	20.7 ± 2.5	0.696
6-mm N subfield	46.4 ± 5.5	48.3 ± 6.1	0.432
3-mm T subfield	17.1 ± 0.8	16.7 ± 0.9	0.243
6-mm T subfield	18.3 ± 1.5	17.8 ± 0.8	0.338
Peripapillary RNFL thickness (µm)			
G	81.9 ± 6.1	84.1 ± 7.9	0.451
N subfield	65.2 ± 7.6	71.4 ± 9.3	0.101
NS subfield	87.0 ± 17.7	95.0 ± 22.0	0.297
TS subfield	119.9 ± 17.4	108.3 ± 25.6	0.216
T subfield	63.9 ± 11.0	60.9 ± 11.0	0.393
TI subfield	129.7 ± 17.3	130.3 ± 13.6	0.926
NI subfield	83.6 ± 12.4	92.9 ± 17.7	0.158

RNFL, retinal nerve fiber layer; GCC, ganglion cell complex; n, number; SD, standard deviation; S: superior; I: inferior; N: nasal; T: temporal; G: central; NS: nasal superior; TS: temporal superior; TI: temporal inferior; NI: nasal inferior.

*Student t test for independent samples.

### OCT-angiography analysis

**Figure 3:**
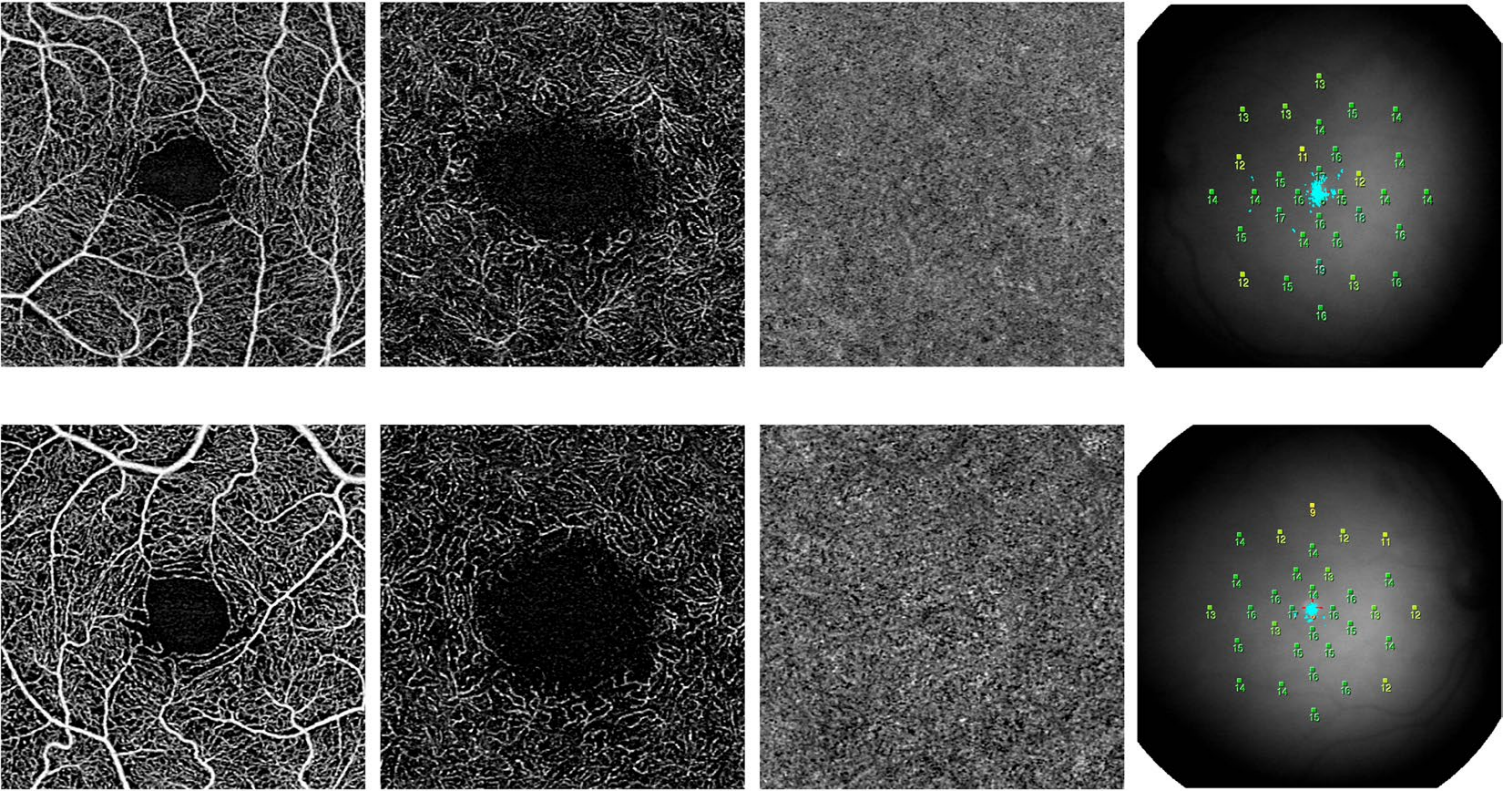
Multimodal imaging in diabetic retinopathy (first row) in comparison with healthy control (second row). (First row) The left eye of a patient with type 1 diabetes without signs of diabetic retinopathy. *En face* optical coherence tomography angiography (OCT-A) images of the superficial capillary plexus (SCP) (first panel), deep vascular complex (DVC) (second panel) and choriocapillaris (third panel) illustrate conserved vessel perfusion. Microperimetry (fourth panel) displays a normal retinal function. (Second row) The right eye of a healthy control. *En face* OCT-A images of the SCP (first panel), DVC (second panel) and choriocapillaris (third panel) illustrate conserved vessel perfusion. Microperimetry (fourth panel) displays a normal retinal function.

Regarding the analysis of retinal plexuses using Swept-Source OCT-A, examinations of the binarized and skeletonized images of patients with type 1 diabetes without complications did not show any microvascular change comparing to control eyes (Fig. 3). In detail, FAZ area was well detectable in all diabetic and control eyes. No significant difference was found in FAZ area by comparing the diabetic and control groups (p = 0.118) (Table 3). Interobserver variability between readers was excellent for FAZ measurements (ICC = 0.991 [0.978–0.996]). Perfusion density of both foveal area and parafoveal area in SCP, DVC, and CC did not show any significant difference between healthy eyes and diabetic one (foveal area: SCP p = 0.808; DVC p = 0.403; CC p = 0.251; parafoveal area: SCP p = 0.114; DVC p = 0.287; CC p = 0.378) (Table 3). No significant difference was also disclosed in VLD for both SCP and DVC between patients with type 1 diabetes without complications and controls in both foveal and parafoveal area (foveal area: SCP p = 0.875; DVC p = 0.196; parafoveal area: SCP p = 0.497, DVC p = 0.838) (Table 3). We also found no significant difference in vessel tortuosity between healthy control and type 1 diabetes without complications groups in both SCP and DVC (p = 0.944 and p = 0.699, respectively).

**Table 3:** Quantitative analysis of FAZ area, perfusion density, vessel length density and vessel tortuosity in 3 × 3 mm OCT-A scans.

	Diabetic eyes (n = 12)	Control eyes (n = 12)	P value*
	*Mean* ± *SD*	*Mean* ± *SD*	
**FAZ area (mm**^**2**^**)**			
Full-thickness retina	0.253 ± 0.059	0.304 ± 0.086	0.118
**Perfusion density**			
*Foveal area*			
SCP	0.315 ± 0.033	0.318 ± 0.028	0.808
DVC	0.064 ± 0.034	0.053 ± 0.024	0.403
CC	0.301 ± 0.027	0.322 ± 0.054	0.251
*Parafoveal area*			
SCP	0.448 ± 0.025	0.462 ± 0.015	0.114
DVC	0.375 ± 0.046	0.397 ± 0.048	0.287
CC	0.261 ± 0.021	0.276 ± 0.053	0.378
**Vessel length density**			
*Foveal area*			
SCP	0.052 ± 0.005	0.052 ± 0.004	0.875
DVC	0.015 ± 0.006	0.012 ± 0.004	0.196
*Parafoveal area*			
SCP	0.068 ± 0.005	0.070 ± 0.005	0.497
DVC	0.064 ± 0.004	0.063 ± 0.010	0.838
**Vessel tortuosity**			
SCP	1.549 ± 0.065	1.547 ± 0.048	0.944
DVC	1.525 ± 0.042	1.537 ± 0.068	0.699

SCP, superficial capillary plexus; DVC, deep vascular complex; CC, choriocapillaris.

*Student t test for independent samples.

The SSI of examinations did not show any significant difference between the two groups (SSI of 8.7 ± 0.9 and 8.4 ± 0.8 in type 1 diabetes without complications group and healthy control). Sex, HTN, patient age, HbA1c level and duration of the disease did not influence significantly the FAZ area, PD and VLD in SCP, DVC and CC (p > 0.05 in all analyses). Furthermore, no significant differences were disclosed between patients under treatment with CSII and with insulin injections with regards to the FAZ area, PD and VLD in SCP, DVC, and CC (p > 0.2 in all analyses).

### Microperimetry analysis

Using microperimetry to test the retinal sensitivity, no differences were disclosed between patients with type 1 diabetes without complications and controls. Mean MS within the central analyzed 10° in the two groups was 11.6 ± 2.4 dB and 12.8 ± 1.36 dB, respectively (p = 0.273). Mean fixation percentage calculated within the central 2° (centered on the fovea) was 93.3 ± 5% in patients with type 1 diabetes without complications and 93.6 ± 3% in controls (p = 0.929), whereas mean fixation percentage within the central 4° (centered on the fovea) was 98.0 ± 2.1% and 98.2 ± 1.2% in patients with type 1 diabetes without complications and controls, respectively (p = 0.625) (Fig. 3). No significant correlation was disclosed between MS with age and HbA1c level (p = 0.172 and p = 0.835, respectively).

## Discussion

In this study, we analyzed the functional and structural features of the retina in patients with type 1 diabetes without signs of DR at fundus examination and systemic vascular complications after 30 years of disease (“happy few” patients with type 1 diabetes without complications). Given that several studies have shown that retinal sub-clinical morphofunctional changes may precede the development of DR in type 1 diabetic patients, we designed this study to clarify whether the “happy few” patients with type 1 diabetes without complications are also characterized by these sub-clinical retinal alterations^3–8,11^.

Using OCT-A, several previous reports have shown evidence of FAZ area enlargement in diabetic eyes without retinopathy compared with normal eyes^3,22^. However, in the present study, the FAZ size did not differ between patients with type 1 diabetes without complications and healthy subjects. According to our results, Carnevali *et al*.^4^ and Meshi *et al*.^23^ found no significant changes in the FAZ size at both SCP and DVC levels in diabetic patients without signs of DR, suggesting that FAZ remodeling and enlargement may appear at a later stage of DR. However, in our study, we considered only a single FAZ of the full-thickness retina, because RNFL, GCC layer and inner plexiform layer (IPL) merge into a single layer near the fovea^24^.

The retinal and CC perfusion was previously studied with OCT-A and a decrease in retinal PD was reported for both type 1 and type 2 DM patients without diabetic retinopathy^3,5,25–31^. In a previous study, Dimitrova *et al*.^32^ reported a decreased PD in the parafoveal area of the SCP and DVC analyzing 29 diabetic eyes in comparison with 33 healthy subjects. Carnevali *et al*.^4^ and our group^21^ also analyzed patients with type 1 DM without any signs of DR and found a reduction of the perfusion density in DVC but not in SCP compared to healthy control subjects. These studies suggested that the retinal capillary density decrease may represent an early process in diabetic eyes and may initially occur in the DVC. In agreement with these results, animal models also showed more vascular abnormalities in the deep vascular network than in the superficial network in diabetic mice^33^. Thus, perfusion density in the DVC may serve as an early and sensitive imaging biomarker of microvascular loss in patients with diabetes.

We add to the literature by reporting the retinal perfusion in a group of “happy few” patients with type 1 diabetes without complications. Interestingly, we did not detect statistically significant differences in SCP and DVC perfusion densities between patients with type 1 diabetes without complications and healthy subjects. Our results differ significantly from the studies mentioned above by suggesting that these patients with long-term T1DM and without evidence or history of systemic and ocular complications do not display any sub-clinical retinal perfusion impairment. In order to provide a comprehensive assessment of the retinal vessels, we further analyzed the VLD and vessel tortuosity. A recent study published by Lee *et al*.^20^ quantified and analyzed the microvascular tortuosity in DR using OCT-A. They found no statistically significant increase of vessel tortuosity in SCP and DVC areas by comparing patients with type 1 diabetes without DR and healthy ones, even if an increased vessel tortuosity was reported in the DR progression^20^. Our data showed that even our study cohort did not show significant differences in vessel tortuosity between healthy and patients with type 1 diabetes without DR.

In our study, we also investigated the choriocapillaris PD and we found no statistically significant differences between the two groups, even if the most recent study realized by Cao and associated^34^ reported that patients with T2DM and no signs of DR showed lower vessel density in SCP, DVC, and CC in comparison with healthy controls.

In summary, our results showed no changes in several OCT-A parameters (FAZ area, PD, VLD, and vessel tortuosity) in both retinal and CC vasculatures between diabetic and control groups.

Furthermore, this study did not find a significant reduction in retinal function and OCT signs of retinal neurodegeneration in our cohort of “happy few” patients with type 1 diabetes without complications.

Several important OCT studies have previously demonstrated that patients with diabetes without DR are characterized by a significant thinning of the inner retinal layers^6,8,35^, which was suggested to be secondary to a progressive ganglion cell and astrocyte loss. Of note, our group demonstrated that sub-clinical vascular alterations assessed with OCT-A precede retinal degeneration, which might, therefore, be secondary to a mechanism of hypoperfusion and ischemia^4^.

Rarely, long-term T1DM patients may be spared by ocular and systemic microvascular complications. Given the rarity of this condition, we referred to these patients with type 1 diabetes without complications as “happy few” subjects. This study displayed that these unusual patients were not characterized by sub-clinical morphofunctional retinal changes. These results may suggest that some mechanisms in patients with diabetes may confer resistance to developing microvascular complications. In the Medalist Study, Keenan and associates^36^ investigated patients affected by insulin-requiring diabetes from more than 50 years, and the authors disclosed that more than 40% of patients did not show signs of significant retinal and renal dysfunction. Since these patients showed no complication despite the long duration of diabetes, the Medalist group appears to be characterized by protective factors, since HbA1c levels were not related to the prevalence of severe diabetic eye and kidney disease. These protective factors could play a role in different pathways related to diabetes, such as the neutralization of toxic effects related to the hyperglycemia and to the mechanisms at the basis of the development and progression of complications related to the disease, and even facilitation of glycemic/metabolic memory^14,37^. Of note, we do exclude that our results depend on low blood glucose levels (mean HbA1c level was 7.3 ± 2.8%) since we previously demonstrated a DVC impairment in post pediatric patients with very similar HbA1c level (mean HbA1c of 7.3 ± 0.7%)^4^.

We acknowledge several limitations of this study. First of all, the relatively small sample size of our series. Furthermore, this study included only Caucasians and then our conclusions may not generalize to people with different origins. Moreover, projection artifacts may also impact on the evaluation of the CC^38^, and, for this reason, we removed from the analysis the CC beneath the major retinal vessels. However, this was a case-control study and thus any potential artifacts may have equally impacted on both disease and control groups. Furthermore, we acquired and analyzed only 3 × 3 mm OCT-A images, and we cannot exclude that more peripheral vascular impairment could be present in these patients.

In conclusion, in a particular population of patients with diabetes who have been spared diabetic complications, newer ophthalmic diagnostic tools confirmed that no retinal microvascular or thickness changes were observed. This idea is very exciting since the finding of these “happy few” patients could help us to better understand and target future treatments for DM and it may allow creating personalized screening intervals providing new perspectives for patient management.

Future long-term follow-up studies are warranted to better understand the evolution of the disease in these patients.

### Disclosures

Riccardo Sacconi, Francesca Lamanna, Enrico Borrelli, Giacomo Mulinacci, Marco Casaluci, Francesco Gelormini, Adriano Carnevali, Lea Querques, Giampaolo Zerbini: none.

Francesco Bandello is a consultant for: Alcon (Fort Worth,Texas,USA), Alimera Sciences (Alpharetta, Georgia, USA), Allergan Inc (Irvine, California,USA), Farmila-Thea (Clermont-Ferrand, France), Bayer Shering-Pharma (Berlin, Germany), Bausch And Lomb (Rochester, New York, USA), Genentech (San Francisco, California, USA), Hoffmann-La-Roche (Basel, Switzerland), Novagali Pharma (Évry, France), Novartis (Basel, Switzerland), Sanofi-Aventis (Paris, France), Thrombogenics (Heverlee,Belgium), Zeiss (Dublin, USA).

Giuseppe Querques is a consultant for: Alimera Sciences (Alpharetta, Georgia, USA), Allergan Inc (Irvine, California, USA), Amgen (Thousand Oaks,USA), Bayer Shering-Pharma (Berlin, Germany), Heidelberg Engineering Inc (Heidelberg, Germany), KBH (Chengdu; China), LEH Pharma (London, UK), Lumithera (Poulsbo; USA), Novartis (Basel, Switzerland), Sandoz (Berlin, Germany), Sifi (Catania, Italy), Sooft-Fidea (Abano, Italy), Zeiss (Dublin, USA).

## Data Availability

The data used to support the findings of this study are available from the corresponding author upon request.

## References

[1] Girach A, Manner D, Porta M (2006). Diabetic microvascular complications: Can patients at risk be identified? A review. Int. J. Clin. Pract..

[2] American Diabetes Association (2018). Classification and Diagnosis of Diabetes: Standards of Medical Care in Diabetes-2018. Diabetes Care.

[3] De Carlo TE (2015). Detection of microvascular changes in eyes of patients with diabetes but not clinical diabetic retinopathy using optical coherence tomography angiography. Retina.

[4] Carnevali A (2017). Optical coherence tomography angiography analysis of retinal vascular plexuses and choriocapillaris in patients with type 1 diabetes without diabetic retinopathy. Acta Diabetol..

[5] Simonett JM (2017). Early microvascular retinal changes in optical coherence tomography angiography in patients with type 1 diabetes mellitus. Acta Ophthalmol..

[6] El-Fayoumi D, Badr Eldine NM, Esmael AF, Ghalwash D, Soliman HM (2016). Retinal Nerve Fiber Layer and Ganglion Cell Complex Thicknesses Are Reduced in Children With Type 1 Diabetes With No Evidence of Vascular Retinopathy. Invest. Ophthalmol. Vis. Sci..

[7] Vujosevic, S. *et al*. Early Microvascular and Neural Changes in Patients With Type 1 and Type 2 Diabetes Mellitus Without Clinical Signs of Diabetic Retinopathy. *Retina*, 10.1097/IAE.0000000000001990 (2017). 10.1097/IAE.0000000000001990 29206758

[8] Vujosevic S, Midena E (2013). Retinal layers changes in human preclinical and early clinical diabetic retinopathy support early retinal neuronal and müller cells alterations. J. Diabetes Res..

[9] Gołębiewska J (2018). Choroidal Thickness and Ganglion Cell Complex in Pubescent Children with Type 1 Diabetes without Diabetic Retinopathy Analyzed by Spectral Domain Optical Coherence Tomography. J. Diabetes Res..

[10] Rohrschneider K, Bültmann S, Springer C (2008). Use of fundus perimetry (microperimetry) to quantify macular sensitivity. Prog. Retin. Eye Res..

[11] Nittala MG, Gella L, Raman R, Sharma T (2012). Measuring retinal sensitivity with the microperimeter in patients with diabetes. Retina.

[12] Montesano G (2017). Structure-function relationship in early diabetic retinopathy: A spatial correlation analysis with OCT and microperimetry. Eye.

[13] Verma A (2009). Is neuronal dysfunction an early sign of diabetic retinopathy? Microperimetry and spectral domain optical coherence tomography (SD-OCT) study in individuals with diabetes, but no diabetic retinopathy. Eye.

[14] Sun JK (2011). Protection from retinopathy and other complications in patients with type 1 diabetes of extreme duration: The Joslin 50-year medalist study. Diabetes Care.

[15] Spaide RF, Curcio CA (2017). Evaluation of Segmentation of the Superficial and Deep Vascular Layers of the Retina by Optical Coherence Tomography Angiography Instruments in Normal Eyes. JAMA Ophthalmol.

[16] Lavia C (2019). Reduced vessel density in the superficial and deep plexuses in diabetic retinopathy is associated with structural changes in corresponding retinal layers. PLoS One.

[17] Sacconi R (2018). Optical coherence tomography angiography in pseudophakic cystoid macular oedema compared to diabetic macular oedema: qualitative and quantitative evaluation of retinal vasculature. Br. J. Ophthalmol..

[18] Sacconi R (2018). Optical coherence tomography angiography in geographic atrophy. Retina.

[19] Sacconi, R. *et al*. Quantitative changes in the ageing choriocapillaris as measured by swept source optical coherence tomography angiography. *Br*. *J*. *Ophthalmol*., 10.1136/bjophthalmol-2018-313004 (2018). 10.1136/bjophthalmol-2018-313004 30361273

[20] Lee H, Lee M, Chung H, Kim HC (2017). Quantification of Retinal Vessel Tortuosity in Diabetic Retinopathy Using Optical Coherence Tomography Angiography. Retina.

[21] Sacconi, R. *et al*. Multimodal Imaging Assessment of Vascular and Neurodegenerative Retinal Alterations in Type 1 Diabetic Patients without Fundoscopic Signs of Diabetic Retinopathy. *J Clin Med*. **8**, 8(9) (2019). 10.3390/jcm8091409 PMC6780853 31500344

[22] Takase N (2015). Enlargement of foveal avascular zone in diabetic eyes evauated by en face optical coherence tomography angiography. Retina.

[23] Meshi, A. *et al*. Anatomical and functional testing in diabetic patients without retinopathy: Results of Optical Coherence Tomography Angiography and Visual Acuity Under Varying Contrast and Luminance Conditions. *Retina*, 10.1097/IAE.0000000000002258 (2018). 10.1097/IAE.0000000000002258 PMC6330253 30015764

[24] Yin X, Chao JR, Wang RK (2014). User-guided segmentation for volumetric retinal optical coherence tomography images. J. Biomed. Opt..

[25] Freiberg FJ (2016). Optical coherence tomography angiography of the foveal avascular zone in diabetic retinopathy. Graefe’s Arch. Clin. Exp. Ophthalmol..

[26] Couturier A (2015). Capillary plexus anomalies in diabetic retinopathy on optical coherence tomography angiography. Retina.

[27] Borrelli, E., Palmieri, M., Viggiano, P., Ferro, G. & Mastropasqua, R. Photoreceptor damage in diabetic choroidopathy. *Retina*, 10.1097/IAE.0000000000002538 (2019). 10.1097/IAE.0000000000002538 30986798

[28] Brunwald JE, DuPont J, Riva CE (1996). Retinal haemodynamics in patients with early diabetes mellitus. Br. J. Ophthalmol..

[29] Alder VA, Su EN, Yu DY, Cringle SJ, Yu PK (1997). Diabetic retinopathy: early functional changes. Clin. Exp. Pharmacol. Physiol..

[30] Liu L (2015). Optical coherence tomography angiography of the peripapillary retina in glaucoma. JAMA ophthalmology.

[31] Yasin Alibhai A (2018). Quantifying microvascular changes using OCT angiography in diabetic eyes without clinical evidence of retinopathy. Ophthalmology Retina.

[32] Dimitrova G (2017). Quantitative Retinal Optical Coherence Tomography Angiography in Patients With Diabetes Without Diabetic Retinopathy. Investig. Opthalmology Vis. Sci..

[33] McLenachan S (2015). Angiography reveals novel features of the retinal vasculature in healthy and diabetic mice. Exp. Eye. Res..

[34] Cao D (2018). Optical coherence tomography angiography discerns preclinical diabetic retinopathy in eyes of patients with type 2 diabetes without clinical diabetic retinopathy. Acta Diabetol..

[35] Picconi F (2017). Retinal neurodegeneration in patients with type 1 diabetes mellitus: the role of glycemic variability. Acta Diabetol..

[36] Keenan HA (2007). Clinical factors associated with resistance to microvascular complications in diabetic patients of extreme disease duration: The 50-year medalist study. Diabetes Care.

[37] Rask-Madsen C, King GL (2013). Vascular complications of diabetes: Mechanisms of injury and protective factors. Cell Metab..

[38] Spaide RF, Fujimoto JG, Waheed NK (2015). Image artifacts in optical coherence tomography angiography. Retina.

